# Correction: Iron application improves yield, economic returns and grain-Fe concentration of mungbean

**DOI:** 10.1371/journal.pone.0232150

**Published:** 2020-04-16

**Authors:** Abdul Majeed, Waqas Ahmed Minhas, Noman Mehboob, Shahid Farooq, Mubshar Hussain, Sardar Alam Cheema, Muhammad Shahid Rizwan

The sixth author's name is spelled incorrectly. The correct name is: Sardar Alam Cheema. The correct citation is: Majeed A, Minhas WA, Mehboob N, Farooq S, Hussain M, Cheema SA, et al. (2020) Iron application improves yield, economic returns and grain-Fe concentration of mungbean. PLoS ONE 15(3): e0230720. https://doi.org/10.1371/journal.pone.0230720
